# Peer victimization as reported by children, teachers, and parents in relation to children's health symptoms

**DOI:** 10.1186/1471-2458-11-278

**Published:** 2011-05-06

**Authors:** Audhild Løhre, Stian Lydersen, Bård Paulsen, Magne Mæhle, Lars J Vatten

**Affiliations:** 1 Department of Public Health, Faculty of Medicine, Norwegian University of Science and Technology, Trondheim, Norway; 2 The Central Norway Regional Health Authority, Stjørdal, Norway; 3 Unit for Applied Clinical Research, Department of Cancer Research and Molecular Medicine, Faculty of Medicine, Norwegian University of Science and Technology, Trondheim, Norway; 4 Sintef Health Center Research, Trondheim, Norway; 5 Center for Child and Adolescent Mental Health, University of Bergen, Norway; 6 Sogn og Fjordane University College, Sogndal, Norway

## Abstract

**Background:**

Victims of bullying in school may experience health problems later in life. We have assessed the prevalence of children's health symptoms according to whether peer victimization was reported by the children, by their teachers, or by their parents.

**Methods:**

In a cross-sectional study of 419 children in grades 1-10 the frequency of peer victimization was reported by children, teachers and parents. Emotional and somatic symptoms (sadness, anxiety, stomach ache, and headache) were reported by the children.

Frequencies of victimization reported by different informants were compared by the marginal homogeneity test for paired ordinal data, concordance between informants by cross-tables and Spearman's rho, and associations of victimization with health symptoms were estimated by logistic regression.

**Results:**

The concordance of peer victimization reported by children, teachers, and parents varied from complete agreement to complete discordance also for the highest frequency (weekly/daily) of victimization. Children's self-reported frequency of victimization was strongly and positively associated with their reports of emotional and somatic symptoms. Frequency of victimization reported by teachers or parents showed similar but weaker associations with the children's health symptoms.

**Conclusion:**

The agreement between children and significant adults in reporting peer victimization was low to moderate, and the associations of reported victimization with the children's self-reported health symptoms varied substantially between informants. It may be useful to assess prospectively the effects of employing different sources of information related to peer victimization.

## Background

Health consequences of peer victimization include higher prevalence of physical complaints and psychosocial maladjustment [1-13], with fairly similar effects between countries [10]. Williams and co-workers drew attention to the importance of dose, and suggested that higher frequencies of victimization were associated with greater risk of health problems [14]. Persistent victimization over an extended time period also predicts more serious health problems [15].

Bullying usually includes aggressive behaviour with repetitive acts and imbalance of power [16,17]. Previous research has shown great variation in bullying and peer victimization among children, and differences in results may partly be due to different research methods [18]. Self-reports of victimization and health outcomes are more common than the use of information from teachers, parents or peers [6], but it has been suggested that using several informants may be useful in assessing effects of victimization [19]. Agreement between different informants about children's health and behavioral problems is typically low to moderate [20-23], but little is known about the concordance between children and significant adults in the assessment of victimization caused by bullying. Also, it is not clear how victimization, as reported by different responders, may be associated with health outcomes.

We therefore tested the consistency of victimization reported by the children, teachers, and parents in a cross-sectional study of school children. Further, we assessed the association of victimization reported by the three sources with the prevalence of emotional and somatic symptoms (anxiety, sadness, stomach ache, headache), as reported by the children.

## Methods

### Participants and procedure

This study is based on a cross-sectional convenience sample of five schools in Møre and Romsdal County, Norway. The headmasters agreed to participate in two surveys that were set two years apart. Since there is no formal agency for ethical approval of school surveys in Norway, the study was approved by the statutory School Collaborative Committees. In the present analysis, data were used from the first survey that was carried out from May to June 2002.

Parents were informed about the survey in the context of a school meeting, and in each class, teachers informed the children about the survey. Information letters signed by the headmaster and by the principal investigator (AL) were sent to all parents, describing the aims of the survey, and emphasising that participation was voluntary and that the collected information was confidential. Children/parents who did not want to participate were asked to notify their main teacher or headmaster. None of the subjects declined to take part in the survey.

Three schools had grades from 1 to 7, and two schools had grades from 1 to 10. All children from four schools and all children in grades 7-10 from the fifth school were included. Thus, 423 children between seven and 16 years of age were invited, together with their parents and teachers. One child moved before the data collection started, and three children were on sick leave during the study period. Thus, 419 (99%) children participated in the study: we received parent responses for 377 (89%) children, and teacher responses for 403 (95%) children.

The questionnaire was developed by the first author (AL), and has demonstrated satisfactory construct, content, and face validity, as described in detail elsewhere [24]. Briefly, reliability of the questionnaire was tested in another material gathered from children in grades 3, 6, and 9. For 179 eligible children, the questionnaire was completed by 154 (86%) children two times, three weeks apart. The test-retest reliability for the 49 ordinal questions was acceptable with 82% of the Spearman's rho coefficients ranging between 0.45 and 0.64 (mean rho = 0.55), and all p-values < 0.001. For the variables used in the present study, the correlations varied from 0.46 to 0.53.

In the current study, the data collection was administered by school nurses and headmasters. Most of the informants filled in the questionnaire themselves, but younger children and children who had problems with reading or writing were interviewed by the school nurses. Thus, 180 children in grades 1-4, 53 children in grades 5-7, and three children in grades 8-10 were interviewed by trained school nurses who used the questionnaire as a guide. Under the instruction of the school nurse or a trained teacher the remaining 183 children completed the questionnaires themselves during a lesson that was allocated to this task. At home, one parent filled in the parents' version of the questionnaire, and the class teacher filled in one questionnaire for each child. The questionnaires from different informants were connected by a specific code.

### Measures

The questionnaires consisted of a combination of items that may promote wellbeing, and items that may adversely affect wellbeing. Victimization (being bullied) was one of the factors that could adversely be associated with good health. Responses to the questions were ranked on ordinal scales, and responses were to be relevant for the current school year. The following items were addressed, each with the corresponding questions:

#### Victimization reported by the children

Three questions were asked: "During recess, are you bothered in some way that makes you feel bad: 1) by being teased, 2) by being hit, kicked, or pushed, 3) by being left out, excluded?". Each question had five response options (1-5): never, seldom, sometimes, about every week, and about every day. In the analyses, we employed the question(s) with the highest response score of the three questions (the max score, i.e. one score only).

#### Victimization reported by parents/teachers

Two questions were asked: "During recess, do others tease or bother your daughter (son) / this child?", and "Does your daughter (son) / this child experience being left out from being together with peers?" Each question had five response options (1-5): never, seldom, sometimes, about every week, and about every day. In the analyses, we employed the question(s) with the highest response score of the two questions (the max score, i.e. one score only).

#### Health symptoms reported by the children

Four questions were asked: "Lately, how often have you felt: 1) sadness, 2) anxiety, 3) stomach ache, 4) headache?" Each question had five response options (1-5): never, seldom, sometimes, often, and always. Sadness and anxiety were denoted emotional symptoms, stomach ache and headache were denoted somatic symptoms.

### Ethics

The survey was approved by the statutory School Collaborative Committees, and the collection of data was approved by The Norwegian Data Inspectorate.

### Statistics

Frequency of victimization as reported by the children, by the parents and by the teachers, was used as the "exposure" variable, and the four health symptoms were "outcome" variables. In the correlation analyses, all five options of victimization were used, whereas in the regression analyses, the last two categories were merged to weekly/daily because of low numbers. Each health symptom was dichotomized into "never/seldom" versus "sometimes/very often/all the time". We used logistic regression analysis to assess the association of victimization frequency with the odds of reporting health symptoms. In the analyses, we adjusted for children's gender and grade in school. Precision of the associations (odds ratios) were assessed using 95% confidence intervals. Correlations were analyzed by Spearman's rho, and paired ordinal data were compared using the marginal homogeneity test. Tests for statistical significance were two-sided, and p-values < 0.05 were considered significant. The statistical analyses were performed in SPSS for Windows (version 18 SPSS, Chicago, Illinois).

## Results

The distribution of the exposure (victimization) and the outcomes (health symptoms) is shown in Table 1. The three informant groups - children, teachers, and parents - reported nearly the same proportions of victimization, but overall, there was a tendency for children to report less victimization than that reported by teachers or parents (p = 0.12 and p = .042, respectively), whereas the reporting of parents and teachers was similar (p = 0.39), marginal homogeneity test. The categories never and seldom victimized (options 1 and 2) were reported for about 80% of the children, sometimes (option 3) was reported for 14-20%, and weekly and daily victimization (options 4 and 5) was reported for 2-4%. Among the children, more than one of four reported to have experienced sadness, stomach ache, or headache sometimes, often, or always (options 3-5), and 17% reported a similar frequency of anxiety.

**Table 1:** The distribution of response options for health symptoms and victimization

Variables	Response options ^a^							
				
	1	2	3	4	5	Total	Mean	SD
				
	%	%	%	%	%	N		
Victimization ^b^	55.2	24.2	16.5	2.2	1.9	417	1.71	0.95
Victimization ^c^	40.5	43.5	13.7	1.0	1.2	402	1.79	0.81
Victimization ^d^	41.1	36.0	19.5	1.9	1.6	375	1.87	0.90
Sadness ^b^	24.5	48.9	23.5	2.7	0.5	413	2.06	0.79
Anxiety ^b^	54.7	28.0	12.9	3.2	1.2	411	1.68	0.90
Stomach ache ^b^	39.6	31.9	21.7	5.1	1.7	414	1.97	0.99
Headache ^b^	38.7	28.5	23.6	7.3	1.9	411	2.05	1.04

^a ^From 1 (never) to 5 (most frequently)

^b ^Reported by children

^c ^Reported by teachers

^d ^Reported by parents

Even though the reported proportions of victimization were fairly similar for the three groups of responders, the concordance in two-way cross-tables of children's responses with those of the respective teacher or parent varied from complete agreement to complete discordance (Table 2). We received responses from 397 child-teacher pairs (Table 2a), from 371 child-parent pairs (Table 2b), and from 359 teacher-parent pairs (Table 2c). The variations in agreement may be exemplified by Table 2a; among 17 children who reported being victims of bullying weekly or daily, teachers reported 10 of these children as being victimized never or rarely, 4 were assigned sometimes, and for 3 children the agreement was complete. Children's reports of being victimized sometimes showed a corresponding concordance with the teachers' reports of victimization. On the other hand, children's reports of being never or seldom victimized (options 1 and 2) were confirmed by teachers in 274 (88%) of the 312 cases and by parents (Table 2b) in 245 (84%) of 292 cases. The estimated correlations (Spearman's rho) between responses were 0.17 for children and teachers, 0.29 for children and parents, and 0.36 for teachers and parents. All correlations were statistically significant (all p-values ≤ 0.001).

**Table 2:** Three cross-tables (2a, 2b, 2c) of victimization (numbers) reported by different informants

2a. Victimization reported by:	Teachers			
	
	1	2	3	4
**Children**				
1: Never	94	99	19	2
2: Seldom	43	38	15	2
3: Sometimes	22	28	16	2
4: Weekly/daily	3	7	4	3


**2b. Victimization** **reported by:**	**Parents**			
	
	1	2	3	4

**Children**				
1: Never	99	76	23	4
2: Seldom	35	35	18	2
3: Sometimes	17	18	25	3
4: Weekly/daily	1	4	7	4


**2c. Victimization** **reported by:**	**Parents**			
	
	1	2	3	4

**Teachers**				
1: Never	78	52	15	0
2: Seldom	59	64	33	2
3: Sometimes	8	13	20	9
4: Weekly/daily	0	1	4	1

We assessed the association of victimization with the prevalence of reported health symptoms, after adjustment for gender and grade. In Table 3, we present associations (odds ratios) of victimization with health symptoms as reported by the children: the results show that a gradual increase in victimization was associated with higher odds of reported health symptoms. Weekly or daily victimization was most strongly associated with both emotional and somatic health symptoms. Compared to never being victimized (the reference category), weekly/daily victimization was associated with approximately seven-fold higher odds of stomach ache (odds ratio, 6.7, 95% CI 2.2 to 20.4) or headache (odds ratio, 7.4, 95% CI 2.4 to 23.0). In relation to emotional symptoms, the corresponding associations with sadness (odds ratio, 3.8, 95% CI 1.3 to 10.8) and anxiety (odds ratio, 5.3, 95% CI 1.7 to 16.1) were also very strong. Less frequent victimization (sometimes) showed statistically significant associations with anxiety, stomach ache and headache (Table 3), and the category seldom victimized was associated with higher odds of anxiety and headache compared to the reference category.

**Table 3:** Associations of children's self-reported peer victimization with their reports of health symptoms

	Health symptoms reported by the children							
	
	Sadness		Anxiety		Stomach ache		Headache	
**Victimization reported by:**	**Odds ratio** **(95% CI)**	**S&V **^**a **^**/ V **^**e**^	**Odds ratio** **(95% CI)**	**A&V **^**b**^**/ V **^**e**^	**Odds ratio** **(95% CI)**	**Sa&V **^**c**^**/ V **^**e**^	**Odds ratio** **(95% CI)**	**Ha&V **^**d**^**/ V **^**e**^
**Children**		**N**		**N**		**N**		**N**

Never	1.00	53/227	1.00	26/226	1.00	51/229	1.00	62/227
Seldom	1.0 (0.5 to 1.7)	23/99	2.0 (1.1 to 3.9)	20/99	1.4 (0.8 to 2.4)	28/99	1.8 (1.1 to 3.1)	37/98
Sometimes	1.6 (0.9 to 3.0)	25/69	3.5 (1.7 to 7.0)	19/68	2.0 (1.1 to 3.6)	26/68	1.9 (1.0 to 3.5)	24/68
Weekly/daily	3.8 (1.3 to 10.8)	9/16	5.3 (1.7 to 16.1)	6/16	6.7 (2.2 to 20.4)	11/16	7.4 (2.4 to 23.0)	11/16

Logistic regression with sadness, anxiety, stomach ache and headache as the dependent variables and victimization as categorical covariate adjusted for gender and grade. The prevalence is stated in numbers (N).

^a ^Sadness & Victimization

^b ^Anxiety & Victimization

^c ^Stomach ache & Victimization

^d ^Headache & Victimization

^e ^Victimization

In separate analyses of boys and girls, the associations of victimization with health symptoms did not substantially differ between the genders, and by dividing the school grades in three groups (1-4, 5-7, and 8-10), the results showed no substantial variation across groups of grades (results not shown).

Similar analyses as those reported in Table 3 were conducted using the frequency of victimization reported by the parents, and by the teachers. Table 4 shows that peer victimization reported by teachers was strongly associated with the children's reported anxiety, but there was no clear association for sadness, or for the somatic symptoms. For victimization reported by parents, each level of victimization was positively associated with anxiety (Table 5), and the highest level of victimization was associated with sadness and stomach ache.

**Table 4:** Associations of teacher-reported peer victimization with health symptoms reported by the children

	Health symptoms reported by the children							
	
	Sadness		Anxiety		Stomach ache		Headache	
	
Victimization reported by:	Odds ratio (95% CI)	**S&V **^**a **^**/ V **^**e**^	Odds ratio (95% CI)	**A&V **^**b**^**/ V **^**e**^	Odds ratio (95% CI)	**Sa&V **^**c**^**/ V **^**e**^	Odds ratio (95% CI)	**Ha&V **^**d**^**/ V **^**e**^
Teachers		N		N		N		N
Never	1.00	46/162	1.00	20/162	1.00	42/163	1.00	45/162
Seldom	0.8 (0.5 to 1.3)	44/168	1.5 (0.8 to 2.8)	28/166	1.1 (0.7 to 1.8)	51/169	1.5 (0.9 to 2.4)	57/168
Sometimes	0.9 (0.4 to 1.8)	14/55	2.9 (1.4 to 6.1)	16/55	1.5 (0.8 to 3.0)	19/54	1.5 (0.8 to 3.0)	20/53
Weekly/daily	1.4 (0.3 to 6.2)	3/8	4.3 (1.0 to 19.5)	3/8	2.5 (0.6 to 10.6)	4/8	1.6 (0.4 to 7.0)	3/8

Logistic regression with sadness, anxiety, stomach ache and headache as the dependent variables and victimization as categorical covariate adjusted for gender and grade. The prevalence is stated in numbers (N).

^a ^Sadness & Victimization

^b ^Anxiety & Victimization

^c ^Stomach ache & Victimization

^d ^Headache & Victimization

^e ^Victimization

**Table 5:** Associations of parent-reported peer victimization with health symptoms reported by the children

	Health symptoms reported by the children							
	
	Sadness		Anxiety		Stomach ache		Headache	
	
Victimization reported by:	Odds ratio (95% CI)	**S&V **^**a **^**/ V **^**e**^	Odds ratio (95% CI)	**A&V **^**b**^**/ V **^**e**^	Odds ratio (95% CI)	**Sa&V **^**c**^**/ V **^**e**^	Odds ratio (95% CI)	**Ha&V **^**d**^**/ V **^**e**^
Parents		N		N		N		N
Never	1.00	37/152	1.00	12/150	1.00	36/152	1.00	61/152
Seldom	1.1 (0.6 to 1.8)	37/133	3.1 (1.4 to 6.7)	24/133	1.5 (0.9 to 2.6)	45/134	0.6 (0.4 to 1.0)	34/133
Sometimes	1.1 (0.6 to 2.1)	21/73	5.3 (2.3 to 12.2)	19/73	1.2 (0.6 to 2.2)	21/71	0.9 (0.5 to 1.7)	24/71
Weekly/daily	3.3 (1.0 to 10.5)	7/13	17.1 (4.7 to 61.7)	7/13	5.0 (1.5 to 16.7)	8/13	2.9 (0.9 to 9.5)	8/13

Logistic regression with sadness, anxiety, stomach ache and headache as the dependent variables and victimization as categorical covariate adjusted for gender and grade. The prevalence is stated in numbers (N).

^a ^Sadness & Victimization

^b ^Anxiety & Victimization

^c ^Stomach ache & Victimization

^d ^Headache & Victimization

^e ^Victimization

## Discussion

In this cross-sectional study of school children, victimization caused by bullying was individually reported by the children, their teacher and their parents, and the concordance between informants was low to moderate. The associations of victimization, as reported by the three sources of information, with the prevalence of emotional and somatic complaints, as reported by the children were also compared. The children's own report of victimization was strongly associated with emotional and somatic complaints, but the reports by teachers and parents showed weaker associations and were mainly related to higher prevalence of anxiety.

The study was conducted in rural communities, ranging from inland to coastal areas. All children attended schools in the Norwegian public school system. The population base and the very high participation are strengths of the study; however, it is a weakness that the data do not include children from urban settings. The convenience sampling of schools and the fact that some schools had grades 1-7 whereas others had grades 1-10 may also be limitations. The reported prevalence of victimization and the consistency of information provided by different informants are, however, in line with findings from other studies. In the collection of data, the younger children were interviewed by school nurses, whereas older children completed the questionnaire themselves. Although the nurses were trained for this task, we cannot exclude the possibility that the different procedures could have influenced the participants and introduced systematic differences in results between younger and older children. The cross-sectional design is a limitation of this study, since cross-sectional designs limit the possibility to study causal effects; the findings must therefore be interpreted with caution.

Self-reports showed that one in five children perceived themselves as being victimized sometimes or more often, and this proportion does not differ substantially from previous findings of Norwegian school children [16,25,26]. Moreover, the prevalence of victimization, as reported by children, teachers, and parents, was fairly similar, although there was a tendency for children to report a lesser degree of victimization. Others have reported differences between informants, where children generally report a higher prevalence of victimization than teachers or parents [27,28].

Only a few studies have assessed the concordance between children's self-reports of victimization and teachers' reports. Of these, Nuijens et al. [29] found no concordance, while other results [19,30] are fairly similar to ours. The agreement - discordance between self-reports and parental reports has rarely been assessed [19], but in a recent such study [31], the estimated consistency for children who reported to be victimized was higher than in our study, but lower for children who perceived themselves not to be victimized. However, only a small proportion of parents (28%) provided information in that study, and the results may be less reliable than our findings.

In line with previous findings [1,6,8], victimization as reported by the children showed strong associations with self-reports of emotional and somatic symptoms. To our awareness, no previous studies have assessed the association of victimization reported by parents with health complaints reported by the children. We found, however, weaker associations of parental reports of victimization with children's self-reported health symptoms compared to children's reports of victimization associated with health symptoms. Victimization as reported by teachers also showed weaker associations with children's health symptoms, and this finding corresponds with results from a recent study [29]. It has been suggested [8] that if exposures and outcomes are simultaneously reported (shared variance), this may result in stronger associations than if the information is derived from different sources. To some degree, this may explain some of the differences in the estimated associations of our study, but the findings call for attention nonetheless.

Further, our results suggest that the frequency of victimization may be particularly important since frequency was positively associated with the prevalence of health symptoms, as reported by the children. In studies where victimization was dichotomized, weak associations have been reported [32], whereas in studies using graded categories of victimization, the associations with health symptoms have typically been stronger among children who report relatively higher frequency of victimization [33]. The effect of dose has also been suggested in a large international study [3]. For all the 28 countries that were included, the symptom load increased with increasing frequency of victimization.

Generally, researchers differ in how they regard the low agreement between respondents [22]. In relation to research on wellbeing and quality of life, comprehensive information from different sources may yield important nuances that may enrich the understanding of children's adjustments [20,21]. In relation to psychopathology, some may prefer to handle information from different sources separately [23,34], whereas others suggest deriving consensus by using information from different perspectives and settings [35,36]. In relation to peer victimization, our results suggest that differences between the sources of information should not be ignored. Children who experience being victimized may be overlooked by significant adults, and these children may at the same time experience high burdens of emotional and somatic symptoms.

## Conclusions

Victims of bullying in school reported high prevalence of sadness, anxiety, stomach ache and headache, and the association with health symptoms showed a strong and graded relation to the frequency of victimization. Children, teachers, and parents reported fairly similar proportions of children to be victimized, but the concordance between informants varied from complete agreement to complete discordance for victimization reported at the highest frequency (weekly/daily). Compared to children's reports, victimization as reported by teachers or parents showed weaker associations with children's self-reported health symptoms. Agreement - discordance among informants should be further assessed, and longitudinal studies may clarify the importance of collecting information on peer victimization from different sources.

## Competing interests

The authors declare that they have no competing interests.

## Authors' contributions

The present cross-sectional study is part of a two year follow-up, planned and administered by AL. All authors participated in designing the study. AL and SL did the analyses. All authors interpreted the data and wrote the paper, and all authors have read and approved the final manuscript.

## Pre-publication history

The pre-publication history for this paper can be accessed here:

http://www.biomedcentral.com/1471-2458/11/278/prepub
